# Commentary: Spacing as the friend of both memory and induction in young and older adults

**DOI:** 10.3389/fnagi.2015.00226

**Published:** 2015-12-15

**Authors:** Nicola Mammarella, Beth Fairfield, Alberto Di Domenico

**Affiliations:** Department of Psychological Sciences, University of ChietiChieti, Italy

**Keywords:** spacing effects, inductive learning, emotion, aging, learning

Inductive learning requires abstracting concepts and categories from examples, that is, learning to generalize examples. The paper by Kornell et al. (2010) examined the influence of spacing or distributed practice on inductive learning in a group of younger and older adults. Their study involved a total of 112 participants, 64 younger adults (55% women, mean age 21) and 48 older adults (56% women, mean age 77). These two groups of participants were asked to learn the styles of 12 different artists. A given artist's paintings were displayed either massed or spaced (that is, interleaved among paintings by the other artists) during study but no painting was ever repeated during study or at test. Spacing was manipulated within participants. After the learning phase, participants were shown paintings by the 12 artists and asked to select the artist who had painted each painting from a list of the artists' names. Results show that inductive learning was better following a spaced presentation rather than a massed presentation and that this finding was independent of age.

Kornell et al. (2010) argue that spacing effectively promoted inductive learning in aging since older adults were able to maintain conceptual memories of a painter's style and consequently form and maintain the sort of gist-based memories that support concept learning via spaced practice. The nature of the materials allowed older adults to engage in encoding processes that support schema abstraction.

Although, this hypothesis is in line with the numerous account of spacing effects in memory, age-related effects could also be explained in terms older adults' disproportionate focus on emotional information relative to non-emotional information and, specifically, on positive rather than negative or neutral information (Di Domenico et al., 2015; Fairfield et al., 2015a,b). Indeed, Mikels et al. (2005) examined age differences in performance on a working memory task in which participants were required to maintain a representation of emotional intensity while they made judgments about pairs of images. Results showed that age-related differences disappeared during the working memory maintenance task in which the to-be-remembered information was emotional. In fact, older adults were able to maintain the emotional intensity and valence of a picture while comparing it to a new picture, especially when dealing with positive pictures. Regardless of the underlying mechanisms, these findings highlight the importance of emotion—cognition interactions in various domains (Mammarella et al., 2012a,b, 2013; Fairfield et al., 2013; Di Domenico et al., 2014). In fact, inductive learning also requires the maintenance of multiple types of mental representations in working memory, including the emotional valence and intensity of a stimulus or event.

It is possible the similar dynamics are going on in Kornell et al.'s (2010) study for two reasons. First, Mammarella et al. (2014) found that it is possible to obtain spacing effects with emotional material, extending the classical learning benefit from spaced practice to emotional learning contexts. Second, viewing a painting usually triggers an emotional response to it. The material used by Kornell et al. (2010) contain landscapes or skyscapes painted by relatively unknown artists. Arguably, when individuals view a painting for the first time they typically assign an emotional connotation to it (I like it/I do not like it) and this may create an efficient basis for inductive learning, especially for older adults. Many pictures from the International Affective Picture System (Lang et al., 2008) which include landscapes or skyscapes are rated as being more positive than other types of content. In Kornell et al.'s (2010) study, older participants may have aided their learning of a painter's style by associating an emotional response to it. However, since the material used in the Kornell et al. (2010)'s study was not previously rated by another independent group of older adults in terms of valence, we cannot exclude this hypothesis. In fact, inductive learning in older adults may have benefited from spaced practice because older adults maintained and compared interleaved paintings by the same painter in terms of the associated valence, intensity or, more in general, emotional connotation.

Another relevant aspect of the study is the fact that following the test phase, participants were asked which type of practice (massive or spaced) helped them learn more. Both younger and older adults expressed a preference for massed practice compared to spaced practice, and this was particularly strong in the older adults: 75% judged massed practice to be more helpful, only 4% thought spacing was superior, and 21% stated that it was about the same. Importantly, these metacognitive data highlighted the illusion that consecutive processing linked to positive emotions (e.g., Berridge and Winkielman, 2003) makes learning easier. In massed presentations, paintings by the same artist were presented consecutively. Participants, and especially older adults, considered it as the best way to abstract the painter's style. These data indicate that the immediate perception of fluency (e.g., the perception of the ease with which the stimulus is processed) generates positive emotions. Consequently, the well-established preference of older adults toward positive emotions reflected in the higher number of massed compared to spaced or other types of responses.

In sum, while Kornell et al. (2010)'s data strongly focus on classical explanations for spacing effects and induction, an interpretation favoring a strong interaction between cognition and emotion cannot be ignored and is highly relevant for directing future research in the area of inductive learning, especially in the aging mind.

## Conflict of interest statement

The authors declare that the research was conducted in the absence of any commercial or financial relationships that could be construed as a potential conflict of interest.

## References

[B1] Berridge K. C. Winkielman P. (2003). What is an unconscious emotion? (The case for unconscious “liking”). Cogn. Emot. 17, 181–211. 10.1080/02699930302289 29715719

[B2] Di Domenico A. Fairfield B. Mammarella N. (2014). Aging and others' pain processing: implications for Hospitalization. Curr. Gerontol. Geriatr. Res. 2014:737291. 10.1155/2014/737291 25254040 PMC4164263

[B3] Di Domenico A. Palumbo R. Mammarella N. Fairfield B. (2015). Aging and emotional expressions: is there a positivity bias during dynamic emotion recognition? Front. Psychol. 6:1130. 10.3389/fpsyg.2015.01130 26300822 PMC4523706

[B4] Fairfield B. Mammarella N. Di Domenico A. (2013). Centenarians' “holy” memory: is being positive enough? J. Genet. Psychol. 174, 42–50. 10.1080/00221325.2011.636399 23534096

[B5] Fairfield B. Mammarella N. Di Domenico A. (2015b). Motivated goal pursuit and working memory: are there age-related differences? Motiv. Emot. 39, 211–215. 10.1007/s11031-014-9428-z

[B6] Fairfield B. Mammarella N. Di Domenico A. Palumbo R. (2015a). Running with emotion: when affective content hampers working memory performance. Int. J. Psychol. 50, 161–164. 10.1002/ijop.12101 25236669

[B7] Kornell N. Castel A. D. Eich T. S. Bjork R. A. (2010). Spacing as the friend of both memory and induction in young and older adults. Psychol. Aging 25, 498–503. 10.1037/a0017807 20545435

[B8] Lang P. J. Bradley M. M. Cuthbert B. N. (2008). International Affective Picture System (IAPS): Affective Ratings of Pictures and Instruction Manual. Technical Report A-8, University of Florida, Gainesville, FL.

[B9] Mammarella N. Fairfield B. De Leonardis V. Carretti B. Borella E. Frisullo E.. (2012a). Is there an affective working memory deficit in patients with chronic schizophrenia? Schizophr. Res. 138, 99–101. 10.1016/j.schres.2012.03.028 22507636

[B10] Mammarella N. Fairfield B. Di Domenico A. (2012b). When touch matters: an affective tactile intervention for older adults. Geriat. Gerontol. Int. 24, 666–673. 10.1111/j.1447-0594.2012.00836.x 22348386

[B11] Mammarella N. Fairfield B. Frisullo E. Di Domenico A. (2013). Saying it with a natural child's voice! When affective auditory manipulations increase working memory in aging. Aging Mental Health 17, 853–862. 10.1080/13607863.2013.790929 23607371

[B12] Mammarella N. Fairfield B. Frisullo E. Di Domenico A. (2014). Does emotion modulate the efficacy of spaced learning in recognition memory? Cogent Psychol. 2014, 1, 986922. 10.1080/23311908.2014.986922

[B13] Mikels J. A. Larkin G. R. Reuter-Lorenz P. A. Carstensen L. L. (2005). Divergent trajectories in the aging mind: changes in working memory for affective versus visual information with age. Psychol. Aging 20, 542–553. 10.1037/0882-7974.20.4.542 16420130 PMC2746384

